# Cost-effectiveness analysis of pembrolizumab versus chemotherapy as first-line treatment for mismatch-repair-deficient (dMMR) or microsatellite-instability-high (MSI-H) advanced or metastatic colorectal cancer from the perspective of the Chinese health-care system

**DOI:** 10.1186/s12913-023-10037-1

**Published:** 2023-10-11

**Authors:** Chen Zhu, Gang Han, Bin Wu

**Affiliations:** 1 Department of Pharmacy, Sir Run Run Shaw Hospital, School of Medicine, Zhejiang University, Hangzhou, 310016 China; 2 Medical Decision and Economic Group, Department of Pharmacy, Ren Ji Hospital, South Campus, School of Medicine, Shanghai Jiaotong University, Shanghai, China

**Keywords:** Pembrolizumab, dMMR, MSI-H, Colorectal cancer, KEYNOTE-177, Cost-effectiveness

## Abstract

**Background:**

Pembrolizumab is superior to chemotherapy as a first-line treatment for patients with mismatch-repair-deficient (dMMR) or microsatellite-instability-high (MSI-H) advanced or metastatic colorectal cancer (CRC), with a significant long-term survival benefit according to the KEYNOTE-177 trial. The current study aimed to determine whether pembrolizumab is a cost-effective treatment for patients with dMMR/MSI-H advanced or metastatic CRC in China.

**Methods:**

A partitioned survival model (PSM) was developed to simulate patients with dMMR/MSI-H advanced or metastatic CRC based on progression-free survival (PFS), progressive disease (PD) and death. The model was designed using a lifetime horizon, a 6-week cycle, and a 5% discount rate. The patients in the model had metastatic dMMR/MSI-H CRC and had not previously received treatment; these characteristics were similar to those of patients in KEYNOTE-177, a phase 3, open-label randomized clinical trial. The health outcomes and utilities were based on the KEYNOTE-177 trial and published data, respectively. Costs were calculated based on local charges (2022) and published literature. A treatment was deemed cost-effective in China if the incremental cost-effectiveness ratio (ICER) value was less than U.S.$38,142.56 per quality-adjusted life-year (QALY). The robustness of the results was assessed via one-way deterministic and probabilistic sensitivity analyses.

**Results:**

Baseline analysis revealed that pembrolizumab provided an additional 2.58 QALYs (3.00 life-year) at an incremental cost of U.S.$78,286.04, resulting in an ICER of U.S.$30,330.15 per QALY, which was below the willingness-to-pay threshold of U.S.$38,142.56 per QALY. When the patient assistance program (PAP) was considered, the ICER became U.S.$1,730.67 per QALY, manifesting absolute cost-effectiveness. The results of sensitivity analyses demonstrated that pembrolizumab was cost-effective in most cases.

**Conclusions:**

Pembrolizumab is a cost-effective first-line treatment for dMMR/MSI-H advanced or metastatic CRC patients in China, especially considering the PAP.

**Supplementary Information:**

The online version contains supplementary material available at 10.1186/s12913-023-10037-1.

## Background

Colorectal cancer (CRC) is one of the most common malignant tumours in China, ranking second in overall cancer incidence and first in digestive tract cancer [1]. In 2020, approximately 550,000 people were diagnosed with CRC, and approximately 280,000 died of CRC, which is second only to lung cancer in China https://gco.iarc.fr/. In addition, the economic burden of CRC continues to grow. The average annual growth rate of medical expenditure per CRC patient in China ranges from 6.9% to 9.2%, and the 1-year out-of-pocket expenditure of a newly diagnosed patient accounts for approximately 60% of their previous-year household income [2–7].

Additionally, more than half of CRC patients are diagnosed at an advanced stage (III—IV) [8, 9] with poor prognosis and distant metastasis, leaving a 5-year overall survival (OS) rate of < 15% [10]. For these patients, standard chemotherapies are 5-fluorouracil (5-FU)-based regimens, such as FOLFOX (5-FU, oxaliplatin, and leucovorin) or FOLFIRI (5-FU, irinotecan, and leucovorin), alone or in combination with therapies that block epidermal growth factor receptor (EGFR) or vascular endothelial growth factor (VEGF) signaling [11]. However, the long-term efficacy of standard chemotherapy is generally poor because among patients with advanced CRC, DNA mismatch-repair-defect (dMMR)/microsatellite-instability-high (MSI-H) CRC accounts for 5%-10% of cases and is associated with resistance to standard chemotherapy and a poor prognosis [12, 13]. Fortunately, with the rapid development of immune checkpoint inhibitors (ICIs) over the past decades, several elegant clinical trials (KEYNOTE-016 [14], KEYNOTE-164 [15], KEYNOTE-177 [16], CheckMate-142 [17]) have shown that durable responses in patients with dMMR/MSI-H metastatic CRC (mCRC) who are refractory to standard chemotherapy combinations can be achieved with programmed death 1 (PD-1) blockade [15, 17–20].

Pembrolizumab was the first PD-1 inhibitor approved by the National Medical Products Administration (NMPA) in China to be adopted as a standard of care first-line treatment for patients with dMMR/MSI-H mCRC based on the data analysis results of a key global phase III clinical trial, namely, KEYNOTE-177 [16]. Recently, the 2021 American Society of Clinical Oncology (ASCO) presented an exciting result that at a median follow-up of more than 44 months (44.5 [36.0–60.3] months in the pembrolizumab group and 44.4 [36.2–58.6] months in the control group), the median OS was not yet achieved in the pembrolizumab group and was 36.7 months in the control group and that the risk of death was reduced by 26% with pembrolizumab treatment (HR, 0.74, 95% CI [0.53–1.03]; *P* = 0.0359). Although PD-1/programmed death-ligand 1 (PD-L1) ICI intervention was administered in 60% of patients in the control group (i.e., doublet chemotherapy ± targeted therapy: either FOLFOX or FOLFIRI, with or without either cetuximab or bevacizumab) after disease progression, the pembrolizumab group still showed a long-term OS benefit. At 36 months, 61% of patients in the pembrolizumab group were still alive, which was 11% higher than that in the control group. In the second interim analysis of KEYNOTE-177 [16], pembrolizumab was found to significantly improve progression-free survival (PFS) compared to the control group as a first-line treatment for dMMR/MSI-H mCRC (median PFS, 16.5 vs. 8.2 months; HR, 0.60; *P* = 0.0002). Superior PFS in the pembrolizumab group remained consistent in subgroup analyses of age, sex, race, region, stage, and BRAF status.

Despite the exciting incremental benefit, the cost of pembrolizumab without national medical insurance is relatively high; thus, the value of this therapy relative to its benefit is unclear. Therefore, we built a partitioned survival model (PSM) to evaluate the cost-effectiveness of pembrolizumab as a first-line regimen in patients with dMMR/MSI-H mCRC from the perspective of the Chinese health-care system with a lifetime horizon.

## Materials and methods

### Model structure

**Fig. 1:**
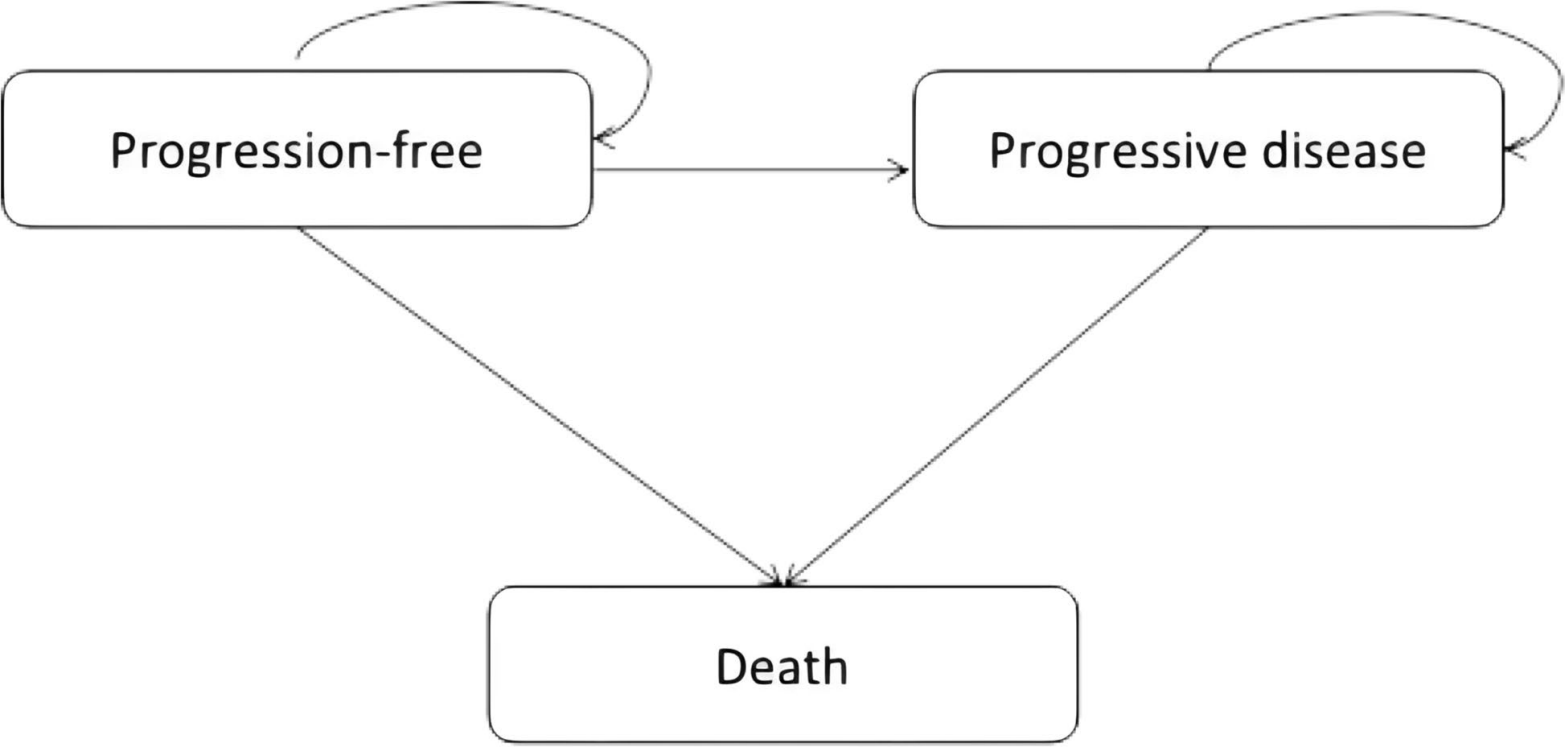
Structure of the partitioned survival mode

To compare the cost-effectiveness of pembrolizumab versus chemotherapy in patients with dMMR/MSI-H stage IV CRC, a PSM was developed to simulate the process of dMMR/MSI-H mCRC depending on the clinical data from KEYNOTE-177. Three distinct health states, PFS, progressive disease (PD) and death (Fig. 1), were included in the model. The initial state was assumed to be PFS, and death was the absorbing state. The population was a cohort with the same characteristics and treatments as those in the KEYNOTE-177 trial. Unlike in the Markov model, the PSM directly used the trials’ Kaplan‒Meier (K-M) curves to divide patients into different health states without making assumptions regarding the transition of patients between different health states. Therefore, the estimation of the proportion of patients in each health state was acquired directly from the cumulative survival probabilities in the OS and PFS curves by parametric function fitting and extrapolation. The cycle length was 6-week (the administration periods of KEYNOTE-177 were different between the experimental group and the control group, namely, 3 weeks and 2 weeks respectively; for the convenience of statistics, we took the lowest common multiple of 3 and 2). The analysis was conducted from the Chinese health-care system perspective with a life-time horizon to ensure that there were fewer than 1% survivors. Costs, life years (LYs), quality-adjusted life years (QALYs), and incremental cost-effectiveness ratios (ICERs) were calculated for each treatment group. If the ICER was below the U.S.$38,142.56 threshold (three times the GDP per capita of China in 2022, ¥262,500.00; the exchange rate of RMB to U.S.$ is 6.9), the treatment was generally considered to be cost-effective. Consistent with Chinese pharmacoeconomic guidelines [21], both costs and benefits were discounted at 5% (range: 0%-8%) per year.

### Efficacy estimates

**Fig. 2:**
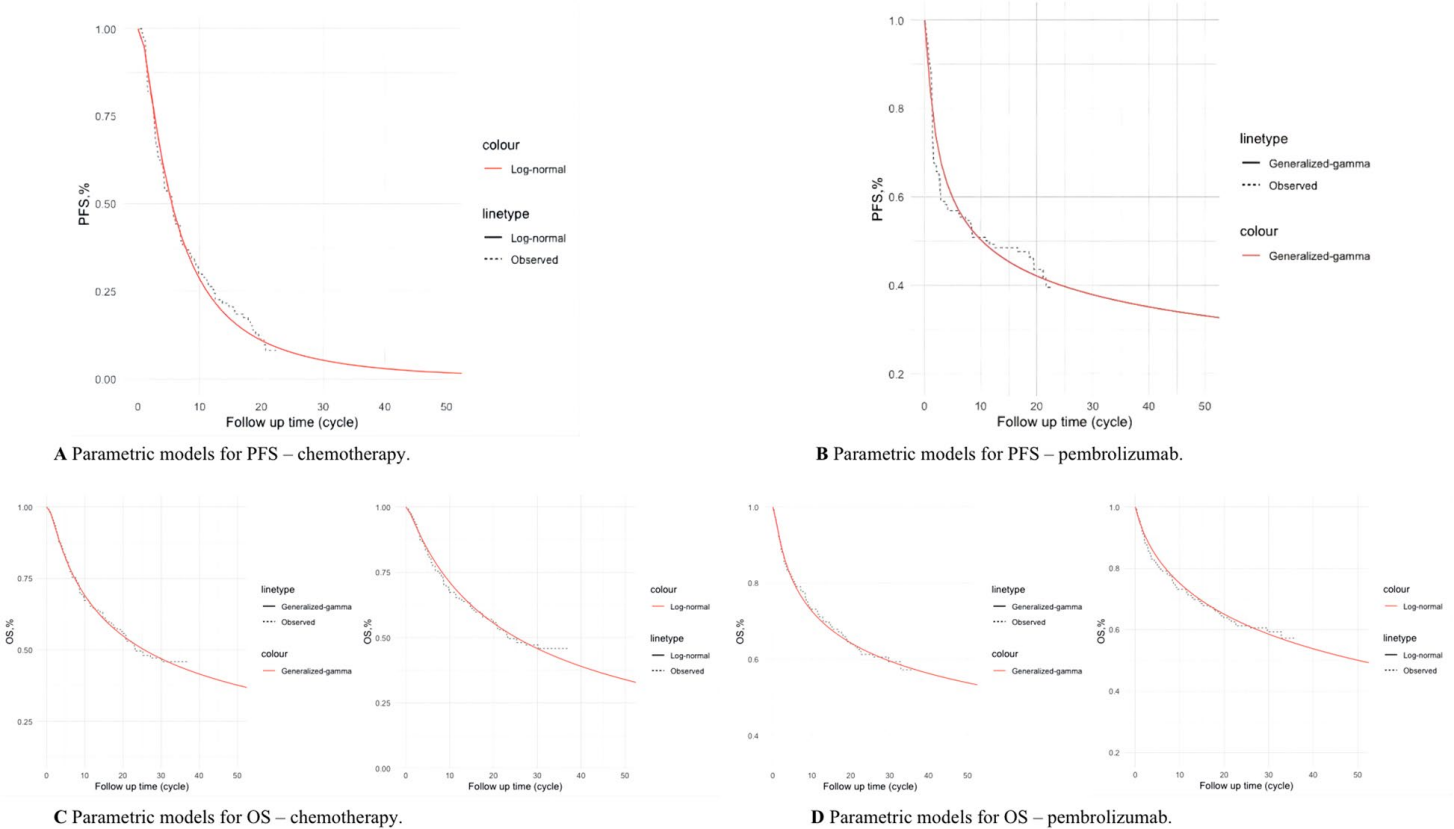
Superposed graphs of the Kaplan‒Meier curves from the KYNOTE-177 trial and the estimated curves. The smooth lines indicate the fitting parametric survival models for intuitively inspecting the survival distributions. OS, overall survival; PFS, progression-free survival

We used Engauge Digitizer (version 12.1, http://digitizer.sourceforge.net) to collect the data points from the K-M curves (PFS and OS curves) of the two arms and followed the method of Guyot et al. [22] to reconstruct estimates of underlying individual patient data (IPD) over the clinical trial time. In terms of the IPD out of the clinical trial time, standard parametric model fitting and extrapolation were used to estimate the long-term survival probabilities by using R software (version 4.1.0, https://www.r-project.org). Specifically, six parametric functions were considered, including the exponential, Weibull, Gompertz, log-logistic, log-normal, and generalized gamma distribution functions. Then, to evaluate the goodness-of-fit of each parametric survival model, multiple methods were applied such as visual inspection, the Akaike information criterion (AIC) test and the Bayesian information criteria (BIC) test proposed in the NICE DSU technical support document 14 (TSD14) [23]. Lower AIC and BIC values indicate a better fit of the selected parametric model (eTables 1–4, provided in Online Resource). Additionally, superposed graphs of the K-M curves from the trial and the estimated curves based on the relatively better fitting parametric survival models are presented in Fig. 2 to intuitively assess the survival prediction.

The generalized gamma model was chosen as the best fit model for the OS curve of both arms and PFS of the pembrolizumab arm, and the log-normal model was chosen for the PFS curve of the chemotherapy arm. Considerations were as follows: 1) the lowest or relatively lower AIC and BIC values among all survival models and 2) the best fit with the observed curves based on visual inspection.

### Utility estimates

The health utility score reflects the level of physical, mental, and social functioning associated with a disease correlative health state that varies from 0 to 1, with 0 representing the worst health state/death and 1 representing the best. The average health utility of the PFS state was 0.7825, which was based on quality-of-life data collected in the KEYNOTE-177 trial [24]. In the trial, the EQ-5D-3L index [25] mean utility scores were 0.77 in the pembrolizumab group and 0.75 in the chemotherapy group at baseline. At the end of week 18, the mean scores were 0.84 and 0.77, respectively. Therefore, in the simulation, we assigned a utility of 0.7825 for PFS, which is the mean of the above values, with 0.75 and 0.84 as the boundaries of the range used in sensitivity analyses. The average health utility of the PD state was 0.64 (95% CI [0.576–0.704]) derived from the previously published literature [26]. The disutility values of grade 3–4 adverse events (AEs) were considered in our analysis [27, 28], but only one-time assessment was carried out during the first cycle for simplification given the trivial influence of AE disutilities. QALY loss caused by AEs was assessed by the product of the incidence of AEs and the corresponding disutility value. In addition, ± 20% were the boundaries of the range in sensitivity analyses.

### Cost inputs

Only direct medical costs were considered, including the cost of the drug utilization, cost of drug administration, cost of follow-up, cost of main AE management and cost of treatments for progression (including active treatments and supportive care), and were stated in 2022 United States dollars (USD) by the following exchange rate: 1 CYN = 0.14 USD. Drug prices were estimated from the local bid-winning price [29].

Drug administration costs were calculated as a function of administration cost per attendance and administration frequency (number of attendances per cycle). Unit administration costs were derived from local charges. The costs of follow-up, including the carcinoembryonic antigen level test, ultrasound of the abdomen, and outpatient specialist clinic, were from a published study [30] and were converted to 2022 USD using the Medical Care component of the Chinese Consumer Price Index (CPI). The frequency of follow-up was based on the guidelines of the Chinese Society of Clinical Oncology (CSCO) for colorectal cancer [11]. The costs of main AE management were derived from the previously published literature [31, 32] (adjusted by CPI) and were calculated only once in the first cycle. In terms of the treatments for progression, active therapeutic schedules included CAPIRI, CAPOX, FOLFOX (± bevacizumab or cetuximab), FOLFIRI (± bevacizumab or cetuximab), nivolumab, and pembrolizumab [33, 34]. The prices of the drugs in subsequent treatments were also from the local bid-winning price [29], and the fee of supportive care was assumed to be 0. Additionally, costs were discounted at an annual rate of 5% [21].

### Clinical inputs

According to the global phase III clinical trial KEYNOTE-177 [16], the patients in the pembrolizumab group (P group) received pembrolizumab at a dose of 200 mg every 3 weeks intravenously (IV) for up to 35 treatments (approximately 2 years), and the patients in the chemotherapy group (C group) received FOLFOX (oxaliplatin 85 mg/m^2^ IV on Day 1, leucovorin 400 mg/m^2^ IV on Day 1, 5-FU 400 mg/m^2^ IV bolus on Day 1 and then 1200 mg/m^2^/day IV over 2 days for a total dose of 2400 mg/m^2^ in each 2-week cycle) or FOLFIRI (irinotecan 180 mg/m^2^ IV on Day 1, leucovorin 400 mg/m^2^ IV on Day 1, 5-FU 400 mg/m^2^ IV bolus on Day 1 and then 1200 mg/m^2^/day IV over 2 days for a total dose of 2400 mg/m^2^ in each 2-week cycle), with or without either cetuximab (cetuximab 400 mg/m^2^ IV over 2 h then 250 mg/m^2^ over 1 h weekly in each 2-week cycle) or bevacizumab (5 mg/kg IV on Day 1 of each 2-week cycle). Treatment was continued until disease progression, development of unacceptable toxic effects, illness, or a decision by the physician or patient to withdraw from the trial. In the C group, the percentages of patients in the mFOLFOX, mFOLFOX + bevacizumab, mFOLFOX + cetuximab, FOLFIRI, FOLFIRI + bevacizumab and FOLFIRI + cetuximab groups were 7.69%, 44.76%, 3.50%, 11.19%, 25.17% and 7.69%, respectively. After disease progression, patients randomly assigned to the C group could crossover to pembrolizumab (to receive a maximum of 35 treatments) and patients in the P group could continue pembrolizumab (to receive a maximum of 17 treatments). The subsequent treatments were mainly composed of pembrolizumab (P group 10%, C group 42.75%), other PD-1/PD-L1 ICIs (P group 7.5%, C group 28.24%), chemotherapy (P group 43.75%, C group 15.27%), VEGF inhibitor (P group 27.5%, C group 9.92%), and EGFR (P group 11.25%, C group 3.82%) inhibitor. Referring to the other studies and guidelines [33, 34], we assumed that CAPIRI, CAPOX, FOLFOX and FOLFIRI were used as the standard second-line chemotherapy, nivolumab (up to 2 years) and pembrolizumab as the ICIs, cetuximab as the EGFR inhibitor and bevacizumab as the VEGF inhibitor, which are commonly used in China. We included grade 3 to 4 AEs in the model that had obvious clinical impact and significantly different rates between the arms of the KEYNOTE-177 trial, which were diarrhoea, anaemia, hypokalaemia, and neutropenia. The incidence of the neutrophil count decrease multiplied by 0.5 was included in the neutropenia assessment. For the dosage calculation, values of body surface area (BSA) and weight were assumed to be 1.80 m^2^ and 65 kg, respectively [35].

### Sensitivity analysis

A series of sensitivity analyses were conducted to test the robustness of the model and address uncertainty in the estimation of model parameters by using Microsoft Excel (version 16.51). One-way deterministic sensitivity analyses (DSAs) were used to evaluate the impact of uncertainty of a single input variable on the ICER. The range of drug prices depends on the local charge or ± 20% of the baseline values. Other parameters were adjusted within the reported 95% confidence intervals (CI) or assuming reasonable ranges of the base case values (± 20%) if 95% CIs were unavailable, in accordance with the established approach [36]. In addition, a separate scenario considering the patient assistance program (PAP) was evaluated in the DSA.

In the probabilistic sensitivity analysis (PSA), a Monte Carlo simulation of 500 iterations was generated by randomly sampling the key model parameters from the prespecified distributions simultaneously. We used a gamma distribution for the cost parameters and a beta distribution for the utility and probability parameters. Based on the data from 500 iterations, a cost-effectiveness acceptability curve (CEAC) was created to represent the likelihood that pembrolizumab would be considered cost-effective compared with chemotherapy on the basis of a willingness to pay (WTP) threshold of U.S.$38,142.56 per QALY in China.

Baseline values, ranges, and distributions for sensitivity analysis are summarized in Table 1.

**Table 1:** Baseline values, ranges, and distributions for sensitivity analysis

Variable	Baseline value	Range		α	β	mean	SE	Reference for baseline value	Distribution (parameters)
		**Minimum**	**Maximum**						
Cycle	6 weeks	-	-	-	-	-	-	-	-
Horizon	Lifetime	-	-	-	-	-	-	-	-
WTP, U.S.$/QALY	38,142.56	-	-	-	-	-	-	[20]	-
Discount rate	0.05	0	0.08	-	-	-	-	[20]	-
Mean body surface area, m^2^	1.80	1.50	1.90	-	-	-	-	[32]	-
Patients’ weight, kg	65	-	-	-	-	-	-	-	-
**Pembrolizumab group _ AE incidence**									
Diarrhea	0.060	0.048	0.072	90.218	1413.409	0.060	0.006	[15]	Beta
Anemia	0.050	0.040	0.060	91.188	1732.572	0.050	0.005		Beta
Hypokalemia	0.010	0.008	0.012	95.070	9411.890	0.010	0.001		Beta
Neutropenia	0.000	0.000	0.000	-	-	0.000	0.000		Beta
**Chemotherapy group _ AE incidence**									
Diarrhea	0.110	0.088	0.132	85.366	690.685	0.110	0.011	[15] Assumed the incidence of neutrophil count decrease multiplied by 0.5 is included in neutropenia	Beta
Anemia	0.100	0.080	0.120	86.336	777.024	0.100	0.010		Beta
Hypokalemia	0.060	0.048	0.072	90.218	1413.409	0.060	0.006		Beta
Neutropenia	0.235	0.188	0.282	73.236	238.405	0.235	0.024		Beta
**Pembrolizumab group second-line therapy proportion**									
Pembrolizumab	0.100	0.080	0.120	86.336	777.024	0.100	0.010	KEYNOTE-177, assumed only the second-line treatments of a proportion of more than 2% were considered in our analysis	Beta
Other PD-1/PD-L1 checkpoint Inhibitor	0.075	0.060	0.090	88.762	1094.731	0.075	0.008		Beta
Chemotherapy	0.438	0.350	0.525	53.585	68.895	0.438	0.045		Beta
VEGF inhibitor	0.275	0.220	0.330	69.354	182.842	0.275	0.028		Beta
EGFR Inhibitor	0.113	0.090	0.135	85.123	671.526	0.113	0.011		Beta
**Chemotherapy group second-line therapy proportion**									
Pembrolizumab	0.427	0.342	0.513	54.557	73.068	0.427	0.044	KEYNOTE-177, assumed only the second-line treatments of a proportion of more than 2% were considered in our analysis	Beta
Other PD-1/PD-L1 checkpoint Inhibitor	0.282	0.226	0.339	68.632	174.362	0.282	0.029		Beta
Chemotherapy	0.153	0.122	0.183	81.225	450.797	0.153	0.016		Beta
VEGF inhibitor	0.099	0.079	0.119	86.410	784.338	0.099	0.010		Beta
EGFR Inhibitor	0.038	0.031	0.046	92.336	2326.872	0.038	0.004		Beta
**Health preferences**									
Utility of PFS	0.7825	0.75	0.84	245.001	63.176	0.795	0.023	[23]	Beta
Utility of PD	0.64	0.576	0.704	137.658	77.432	0.640	0.033	[25]	Beta
Disutility due to AEs (grade ≥ 3)									
Diarrhea	-0.090	-0.072	-0.108	14.470	146.308	-0.090	0.023	[26, 27]	Beta
Anemia	-0.085	-0.068	-0.102	14.555	156.680	-0.085	0.021		Beta
Hypokalemia	-0.080	-0.064	-0.096	14.640	168.360	-0.080	0.020		Beta
Neutropenia	-0.0607	-0.049	-0.073	14.968	231.623	-0.061	0.015		Beta
**Drug cost, U.S.$/mg**									
Pembrolizumab	179.18	-	-	-	-	-	-	2022 Local charge https://www.yaozh.com	Fix
Oxaliplatin	3.4	2.72	4.08	16.000	0.213	3.400	0.850		Gamma
Leucovorin	0.25	0.2	0.3	16.000	0.016	0.250	0.063		Gamma
5-FU	0.29	0.232	0.348	16.000	0.018	0.290	0.073		Gamma
Bevacizumab	15	11.88	15	16.000	0.840	13.440	3.360		Gamma
Cetuximab	12.04	-	-	-	-	-	-		Fix
Irinotecan	17.73	14.05	24.9	16.000	1.217	19.475	4.869		Gamma
Nivolumab	96.2	-	-	-	-	-	-		Fix
Capecitabine	0.04	0.01	0.04	16.000	0.002	0.025	0.006		Gamma
**AE cost, U.S.$**									
Diarrhea	392.44	313.95	470.93	16.000	24.527	392.439	98.110	[31]	Gamma
Anemia	724.64	579.71	869.57	16.000	45.290	724.638	181.160	[30]	Gamma
Hypokalemia	157.28	125.82	188.73	16.000	9.830	157.275	39.319	[30], assumed same as fatigue	Gamma
Neutropenia	628.96	503.17	754.76	16.000	39.310	628.965	157.241	[30]	Gamma
**Administration, U.S.$/attendance**	310.16	248.13	372.19	16.000	19.385	310.160	77.540	Local charge	Gamma
**Cost of BSC, U.S.$/cycle**	0.00	0.00	0.00	-	-	-	-	Assumed to be 0	-
**Follow up, U.S.$/cycle**	31.33	25.06	37.60	16.000	1.958	31.330	7.833	[10, 29]	Gamma

*PD-1* programmed death 1, *PD-L1* programmed death ligand 1, *EGFR* epidermal growth factor receptor, *VEGF* vascular endothelial growth factor, *PD* progressive disease, *PFS* progression-free survival, *AE* adverse event, *BSC* best supportive care

## Results

### Base case results

The base case model results are listed in Table 2. Based on the reconstructed estimates of IPD, we found that the probabilities of PFS and PD (P group vs. C group) in the first year were 55.0% vs. 40.9% and 21.9% vs. 35.0%, respectively, while in the second year, there was a more obvious gap between the two groups (45.3% vs. 17.0% and 22.4% vs. 43.7%, respectively). Over a lifetime horizon, the use of pembrolizumab compared with chemotherapy produced a gain of an extra 3.00 LYs, which was also reflected in the superposed graph of simulated survival curves (OS) for chemotherapy and pembrolizumab (eFigure 1 in the supplement). When adjusted for quality of life, the total QALYs for pembrolizumab and chemotherapy were estimated to be 6.71 and 4.13, respectively. According to the dosage scheme used, the total costs based on the PSM were U.S.$229,698.49 and U.S.$151,412.44, respectively, during that period, resulting in an ICER of U.S.$30,330.15 per QALY.

**Table 2:** Base-case results

Strategy	Chemotherapy	Pembrolizumab
Cost (U.S.$)	151,412.44	229,698.49
LYs	6.22	9.22
QALYs	4.13	6.71
Incremental cost (U.S.$)^a^	NA	78,286.04
Incremental LYs^a^	NA	3.00
Incremental QALYs^a^	NA	2.58
Incremental cost per LY gained^a^	NA	**$8,284.72**
Incremental cost per QALY gained^a^	NA	**$30,330.15**

*LYs* life years, *QALYs* quality-adjusted life years

^a^Compared with chemotherapy

### Sensitivity analysis

#### Results of the deterministic sensitivity analysis

**Fig. 3:**
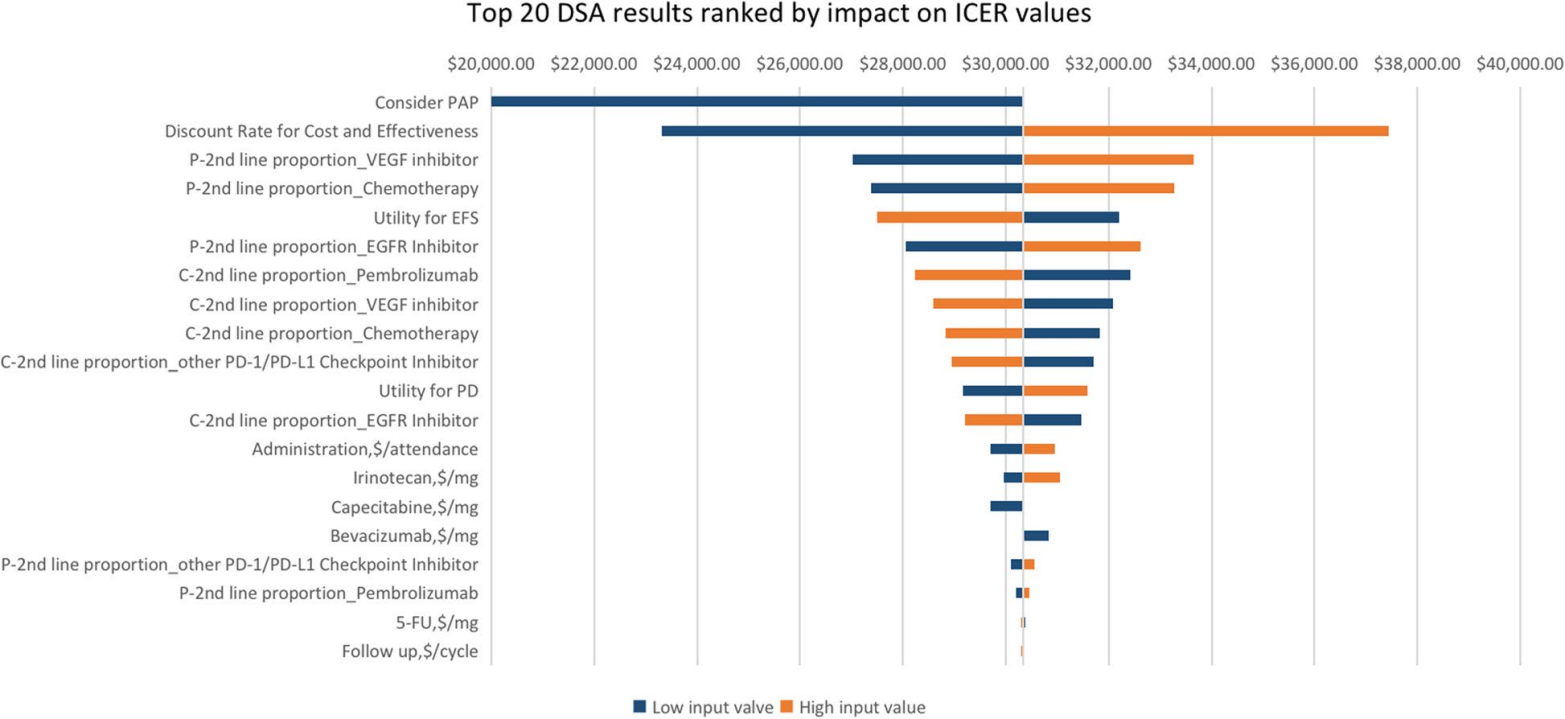
Top 20 one-way deterministic sensitivity analysis (DSA) results ranked by impact on ICER values. Tornado diagrams show the influence of factors on the partitioned survival model of the two strategies in the treatment of mCRC. The factors are listed in descending order of their influence on ICER with variation in the factor values. ICER, incremental cost-effectiveness ratio; PD, progressive disease; PFS, progression-free survival; PAP, patient assistance program; P, pembrolizumab; C, chemotherapy

The DSA results are presented in the tornado diagram (Fig. 3). The parameters with the greatest influence on the ICER were consideration of PAP, discount rate for cost and effectiveness, proportion of subsequent treatments, and baseline utility value. Across arbitrary changes in the ranges for each parameter, the ICER remained < U.S.$38,142.56 per QALY, except that the discount rate became 0.08 and the proportion of VEGF inhibitors used as a 2^nd^-line regimen in the pembrolizumab group increased by 20%. The incidence, cost, and disutility for AEs had a tiny impact on the ICER. In addition, when PAP was considered, the ICER became U.S.$1,730.67 per QALY, manifesting absolute cost-effectiveness.

#### Results of the probabilistic sensitivity analysis

**Fig. 4:**
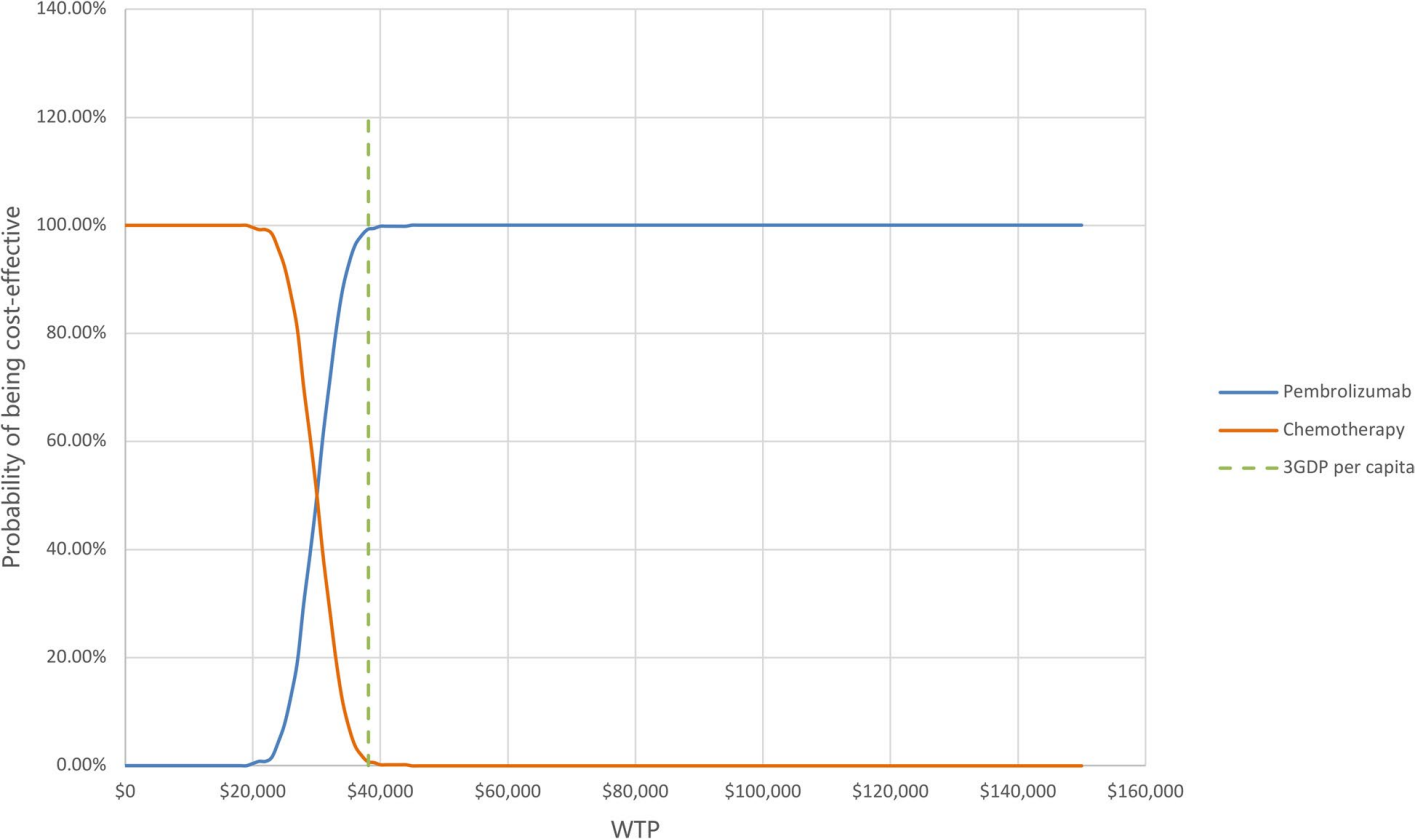
Cost-effectiveness acceptability curves (CEACs). The cost-effectiveness acceptability frontier shows the probability of two strategies being cost-effective. WTP, willingness-to-pay; P, pembrolizumab; C, chemotherapy; 3GDP, 3-times estimated per capita GDP of China

**Fig. 5:**
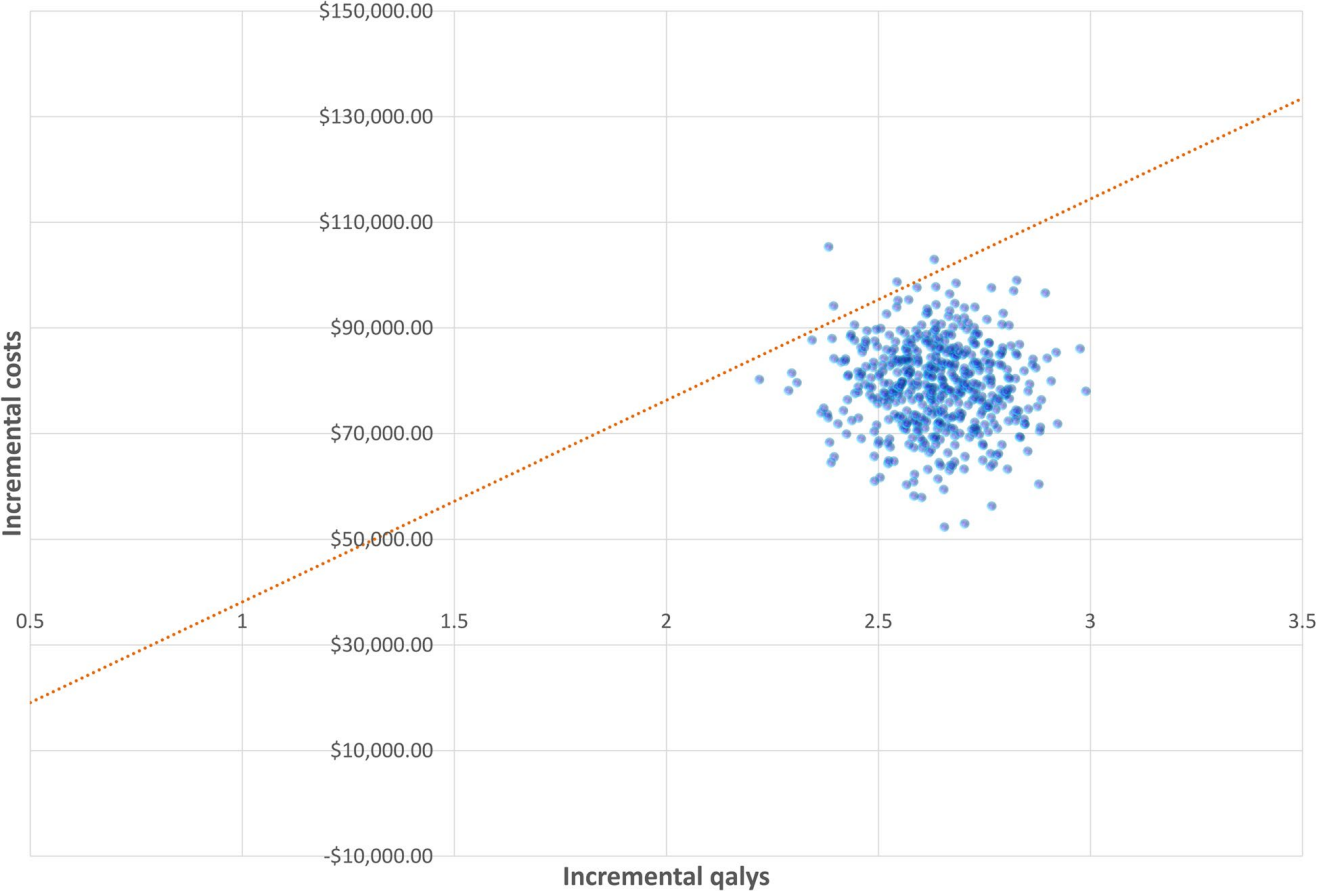
Incremental cost-effectiveness scatter plot distribution (WTP = $38,142.56). Probabilistic sensitivity analysis was performed to test the robustness of the model and address uncertainty in the estimation of model parameters by using 500 Monte Carlo simulations. Most dots were under the WTP threshold ($38,142.56 per QALY), indicating that pembrolizumab probably generated more QALYs with acceptable incremental costs. QALY, quality-adjusted life-year; WTP, willingness-to-pay

The PSA results are shown in the CEAC in Fig. 4, which shows the probability that pembrolizumab is cost-effective along with an increase in WTP values. These outcomes indicated a nearly 0% probability that pembrolizumab was cost-effective at WTP values of U.S.$20,000 per QALY. There were 50% and 100% opportunities that pembrolizumab was cost-effective at WTP values of approximately U.S.$30,000 and U.S.$45,000 per QALY, respectively. The scatter plot shown in Fig. 5 depicts the results of the 500 simulations of the PSA, in which, most results were under the WTP threshold (U.S.$38,142.56 per QALY), indicating that pembrolizumab probably generated more QALYs with acceptable incremental costs.

## Discussion

### Summary and interpretation of results

5-FU in combination with either oxaliplatin or irinotecan (± VEGF or EGFR inhibitor) is an established treatment for mCRC, with good clinical efficacy at an acceptable cost [14, 37–45]. With the dramatic development of ICIs over the past decades, some clinical trials [14–17] have indicated that PD-1 blockade used alone, such as pembrolizumab and nivolumab approved by NMPA or FDA, has achieved much more effectiveness in patients with dMMR/MSI-H metastatic or unresectable CRC that is resistant to the standard chemotherapy combinations on a longer course of treatment [15, 17–20]. A study [46] suggested that increased tumour grading (captured by Ki-67) is associated with impairment of antitumour immunity through HLA-I downregulation, reduced CD8 infiltration and enhanced PD-L1/PD-1 expression on tumour cells. Therefore, it may be possible to predict the efficacy of ICIs by detecting the expression of Ki-67. However, these PD-1 blockade therapies have significant costs, and their value remains unclear. Cost-effectiveness analyses provide a feasible methodology for evaluating the cost-effectiveness of a regimen, based on the survival benefit, quality of life, costs of administration and drugs, costs of follow-up and costs of AEs. However, at present, there is no cost-effectiveness analysis of pembrolizumab or nivolumab used alone in patients with dMMR/MSI-H mCRC.

Pembrolizumab was the first and only PD-1 inhibitor approved by the NMPA in China as the first-line therapy for patients with dMMR/MSI-H mCRC based on the results of the KEYNOTE-177 trial. The trial demonstrated that pembrolizumab treatment significantly prolonged survival compared with standard chemotherapy. In the Chinese context, given the incremental benefit and cost related to this treatment, we conducted the first study, to our knowledge, detecting the cost-effectiveness of pembrolizumab as the first-line regimen in patients with dMMR/MSI-H advanced or metastatic CRC. On the basis of our model, pembrolizumab was projected to extend patient life expectancy to a point not previously seen in this patient population. For instance, in the pembrolizumab arm, life expectancy was projected to reach nearly 9 years, approximately 1.5 times that of the chemotherapy group (increase patients’ survival by 3 years with discounting), despite the high proportion of crossover in the control group after disease progression. In addition, from the perspective of safety, the incidence of AEs (Grade ≥ 3), such as diarrhoea, anaemia, hypokalaemia and neutropenia, in Group P was lower than that in Group C, which, to some extent, reduced the cost and improved the quality of life for patients. The associated incremental cost per QALY gained was U.S.$30,330.15. While there is no single established ICER threshold for cost-effectiveness in China, the World Health Organization has referenced a threshold of 3 times the estimated per capita GDP with respect to disability-adjusted life years, which in China for in 2022 would correspond to U.S.$38,142.56/QALY [47]. The ICER in the basic analysis was below this threshold, suggesting that pembrolizumab monotherapy could be selected as a cost-effective first-line treatment for patients with dMMR/MSI-H advanced or metastatic CRC in clinical practice. However, the threshold for a minimum important change (MIC) was 0.06–0.09 points for the EQ-5D-3L health utility score [24]. When considering the point of view of patients measured by the MIC, the increased health-related quality of life (HRQOL) in our study (P group 0.73 vs. C group 0.66) could be negligible. This result might be influenced by the high proportion of crossover in the control group after disease progression.

In univariable sensitivity analyses, the most influential driver of the ICER was the consideration of PAP. The PAP called “Key to Life” was approved in China on July 8, 2021 [48], officially providing support that alleviates patients’ out-of-pocket expenses for pembrolizumab (as the first-line monotherapy) for dMMR/MSI-H metastatic or unresectable CRC patients with wild-type KRAS, NRAS and BRAF genes. Based on this assistance program, it only costs U.S.$11,200 per year (up to 2 years, U.S.$22,400) with a decrease of approximately 88.8%. Considering that, we conducted a scenario analysis and found that the basic ICER became U.S.$1,730.67 per QALY, which indicated that the patients could receive more benefits with minimal incremental costs. Under such circumstances, we fully recommend that mCRC patients who meet the abovementioned conditions be administered pembrolizumab monotherapy as the first-line regimen. The impact of the discount rate on the basic results ranked second. In addition, due to the relatively high price of biological agents [29], the proportion of VEGF/EGFR inhibitors used as 2^nd^-line regimens also had a great effect on the outcomes. For this, some scholars [49, 50] have found that treatment with bevacizumab in Chinese patients with mCRC is unlikely to use financial resources efficiently. In terms of EGFR inhibitors (cetuximab), Wu, B. et al. [51] and Wang, H. et al. [52] have conducted cost-effective analyses based on the CRYSTAL trial [40] and the TAILOR trial [53] and found that cetuximab combined with FOLFOX or FOLFIRI was not a cost-effective treatment for the patients with RAS wild-type mCRC in China unless the PAP was available. These two analyses were performed in 2017 and 2020, respectively, which might not be comparable with the current situation. However, in the past two years, the increase in the medical care component of CPI in China was small (0.4% and 0.6%), while the price of cetuximab remained unchanged; therefore, the ICER from Wang, H. et al. [52] could be referenced. In addition, although per capita GDP has been increasing, according to the CEAC in the study (Wang, H. et al.), we found that cetuximab was still not cost-effective in China under the WTP of 2022. When varying all model parameters in 500 Monte Carlo simulations, the PSA indicated a more than 99.2% probability that pembrolizumab would be considered cost-effective at a usually accepted value (U.S.$38,142.56 per QALY). In China, drugs are purchased and priced by the state in a centralized way. Therefore, drug prices in all provinces and regions are basically identical. According to the PSA (Fig. 4), each province and region can determine whether pembrolizumab is cost-effective based on its own per capita GDP.

### Strengths and limitations

A major strength of the basic analysis was its dependence on a direct comparison of pembrolizumab and standard chemotherapy, utilizing data and information from a randomized controlled trial. In addition, the PSM structure is unnecessary for building assumptions for the transition probabilities of patients but has the advantage of being able to partition patients to different health states directly based on the trial’s K-M curves. Moreover, the time dependence of risk can be handled automatically instead of applying constant transition risks within a traditional Markov model.

It is essential to note that our study had several limitations. First, the development of the cost-effectiveness analysis was based on the results of the KEYNOTE-177 trial, in which the participants were mainly from the Western Europe or North America (> 70%). To some extent, this may impair the application of our research results. Second, the values of utilities of PFS and PD were derived from the study on the health-related quality of life in the trial population (the sample size might not be sufficient to reflect the true utilities), and the disutilities of AEs were derived from previously published studies, which might not reflect the health state of patients in China. This defect may also affect the robustness of our results. Updated health utility data for patients with dMMR/MSI-H metastatic or unresectable CRC in Chinese populations might enhance the accuracy and robustness of the analyses. Third, the efficacy data of PFS and the incidence of AEs were from the interim results of the trial because the final results have not been officially published. Thus, it is possible that projections of cost-effectiveness could change with additional follow-up. Fourth, without considering patient compliance, the cost of follow-up may be overestimated. Because of this, the cost of follow-up as a variable was included in the DSA, and we found that the impact of the follow-up cost on the results was small. In addition, for the longer-term extrapolation of PFS and OS, there existed an inherent uncertainty, and the perspective of the Chinese health-care system may limit the scope of application of the results. Finally, due to the research perspective, our study only included direct medical costs. If indirect costs were added, the advantage of Group P might be more significant as disease improvement could reduce indirect costs.

## Conclusion

From the perspective of the Chinese health-care system, the current model predicted that pembrolizumab monotherapy is more likely to be a cost-effective first-line strategy for patients with dMMR/MSI-H advanced or metastatic CRC. When PAP is available, pembrolizumab monotherapy is the dominant treatment strategy. The reported conclusions may be helpful to physicians, mCRC patients and health management agencies in their decision-making processes.

### Supplementary Information

**Additional file 1: eTable 1.** Summary of goodness of fit statistics for pembrolizumab – OS. **eTable 2.** Summary of goodness of fit statistics for pembrolizumab – PFS. **eTable 3.** Summary of goodness of fit statistics for chemotherapy – OS. **eTable 4.** Summary of goodness of fit statistics for chemotherapy – PFS. **eFigure 1.** Simulated survival curves (OS) for chemotherapy and pembrolizumab.

## Data Availability

Most data generated or analyzed during this study are included in this published article (and its supplementary information files). Complete datasets are available from the corresponding author on reasonable request.

## Abbreviations

CRC: Colorectal cancer
OS: Overall survival
5-FU: 5-Fluorouracil
FOLFOX: 5-FU, oxaliplatin, and leucovorin
FOLFIRI: 5-FU, irinotecan, and leucovorin
EGFR: Epidermal growth factor receptor
VEGF: Vascular endothelial growth factor
MSI-H: Mismatch repair defect (dMMR) /microsatellite instability high
ICIs: Immune checkpoint inhibitors
PD-1: Programmed death 1
PD-L1: Programmed death ligand 1
mCRC: Metastatic CRC
NMPA: National Medical Products Administration
ASCO: American Society of Clinical Oncology
PFS: Progression-free survival
PSM: Partitioned-survival model
PD: Progressive disease
K-M: Kaplan–Meier
LYs: Life years
QALYs: Quality-adjusted life years
ICERs: Incremental cost-effectiveness ratios
IPD: Individual patient data
AIC: Akaike information criterion
BIC: Bayesian information criteria
AEs: Adverse events
USD: United States dollars
CPI: Consumer Price Index
CSCO: Chinese Society of Clinical Oncology
IV: Intravenously
BSA: Body surface area
DSA: Deterministic sensitivity analysis
CI: Confidence interval
PAP: Patient assistance program
PSA: Probabilistic sensitivity analysis
CEAC: Cost-effectiveness acceptability curve
WTP: Willingness to pay
