# Supplementary material for: Cost-effectiveness analysis of pembrolizumab versus chemotherapy as first-line treatment for mismatch-repair-deficient (dMMR) or microsatellite-instability-high (MSI-H) advanced or metastatic colorectal cancer from the perspective of the Chinese health-care system
Source: BMC Health Serv Res. 2023 Oct 11;23:1083. doi: 10.1186/s12913-023-10037-1 (PMC10568806; doi:10.1186/s12913-023-10037-1)
Supplement: Supplementary file 1 — Additional file 1: eTable 1. Summary of goodness of fit statistics for pembrolizumab – OS. eTable 2. Summary of goodness of fit statistics for pembrolizumab – PFS. eTable 3. Summary of goodness of fit statistics for chemotherapy – OS. eTable 4. Summary of goodness of fit statistics for chemotherapy – PFS. eFigure 1. Simulated survival curves (OS) for chemotherapy and pembrolizumab. [file 12913_2023_10037_MOESM1_ESM.docx]

Cost-effectiveness analysis of pembrolizumab versus chemotherapy as the first-line treatment for patients with mismatch-repair-deficient (dMMR) or microsatellite- nstability-high (MSI-H) advanced or metastatic colorectal cancer from the perspective of the Chinese health-care system

BMC Health Services Research

Chen Zhu^1^, Gang Han^1^, Bin Wu^2^

1.Department of Pharmacy, Sir Run Run Shaw Hospital, School of Medicine, Zhejiang University, Hangzhou 310016, China

2.Medical Decision and Economic Group, Department of Pharmacy, Ren Ji Hospital, South Campus, School of Medicine, Shanghai Jiaotong University, Shanghai, China.

Chen Zhu, E-mail: [zc15011553821@163.com](mailto:zc15011553821@163.com)

Correspondence author: Gang Han, E-mail: [3199022@zju.edu.cn](mailto:3199022@zju.edu.cn)[,](mailto:hanpharm@163.com,) Bin Wu, E-mail: [scilwsjtu-wb@yahoo.com](mailto:scilwsjtu-wb@yahoo.com)

*Supplement*

*eTable 1. Summary of goodness of fit statistics for pembrolizumab - OS*

| *Treatment* | *Efficacy inputs* | *Parametric function* | *AIC* | *BIC* |
| --- | --- | --- | --- | --- |
| Pembrolizumab | OS | Exponential | 618.3963 | 621.4267 |
|  |  | Weibull | 602.6328 | 608.6937 |
|  |  | Log-logistic | 599.8584 | 605.9192 |
|  |  | Log-normal | 595.7375 | *601.7984* |
|  |  | Gompertz | 596.7492 | 602.8101 |
|  |  | *Generalized gamma* | *593.5697* | 602.6610 |
| Abbreviations: OS, overall survival; AIC, Akaike information criterion; BIC, Bayesian information criterion | | | | |

*eTable 2. Summary of goodness of fit statistics for pembrolizumab - PFS*

| *Treatment* | *Efficacy inputs* | *Parametric function* | *AIC* | *BIC* |
| --- | --- | --- | --- | --- |
| Pembrolizumab | PFS | Exponential | 639.9757 | 643.0061 |
|  |  | Weibull | 599.5594 | 605.6203 |
|  |  | Log-logistic | 588.7002 | 594.7611 |
|  |  | Log-normal | 581.1180 | 587.1789 |
|  |  | Gompertz | 579.0181 | 585.0790 |
|  |  | *Generalized gamma* | *565.3594* | *574.4507* |
| Abbreviations: PFS, progression-free survival; AIC, Akaike information criterion; BIC, Bayesian information criterion | | | | |

*eTable 3. Summary of goodness of fit statistics for chemotherapy - OS*

| *Treatment* | *Efficacy inputs* | *Parametric function* | *AIC* | *BIC* |
| --- | --- | --- | --- | --- |
| Chemotherapy | OS | Exponential | 729.9633 | 732.9938 |
|  |  | Weibull | 728.9004 | 734.9613 |
|  |  | Log-logistic | 723.7526 | 729.8134 |
|  |  | Log-normal | 719.6843 | *725.7452* |
|  |  | Gompertz | 722.1885 | 728.2494 |
|  |  | *Generalized gamma* | *719.1524* | 728.2437 |
| Abbreviations: OS, overall survival; AIC, Akaike information criterion; BIC, Bayesian information criterion | | | | |

*eTable 4. Summary of goodness of fit statistics for chemotherapy - PFS*

| *Treatment* | *Efficacy inputs* | *Parametric function* | *AIC* | *BIC* |
| --- | --- | --- | --- | --- |
| Chemotherapy | PFS | Exponential | 724.9124 | 727.9493 |
|  |  | Weibull | 725.6351 | 731.7090 |
|  |  | Log-logistic | 714.5306 | 720.6045 |
|  |  | *Log-normal* | *708.3561* | *714.4301* |
|  |  | Gompertz | 726.7112 | 732.7851 |
|  |  | Generalized gamma | 708.6698 | 716.7806 |
| Abbreviations: PFS, progression-free survival; AIC, Akaike information criterion; BIC, Bayesian information criterion | | | | |


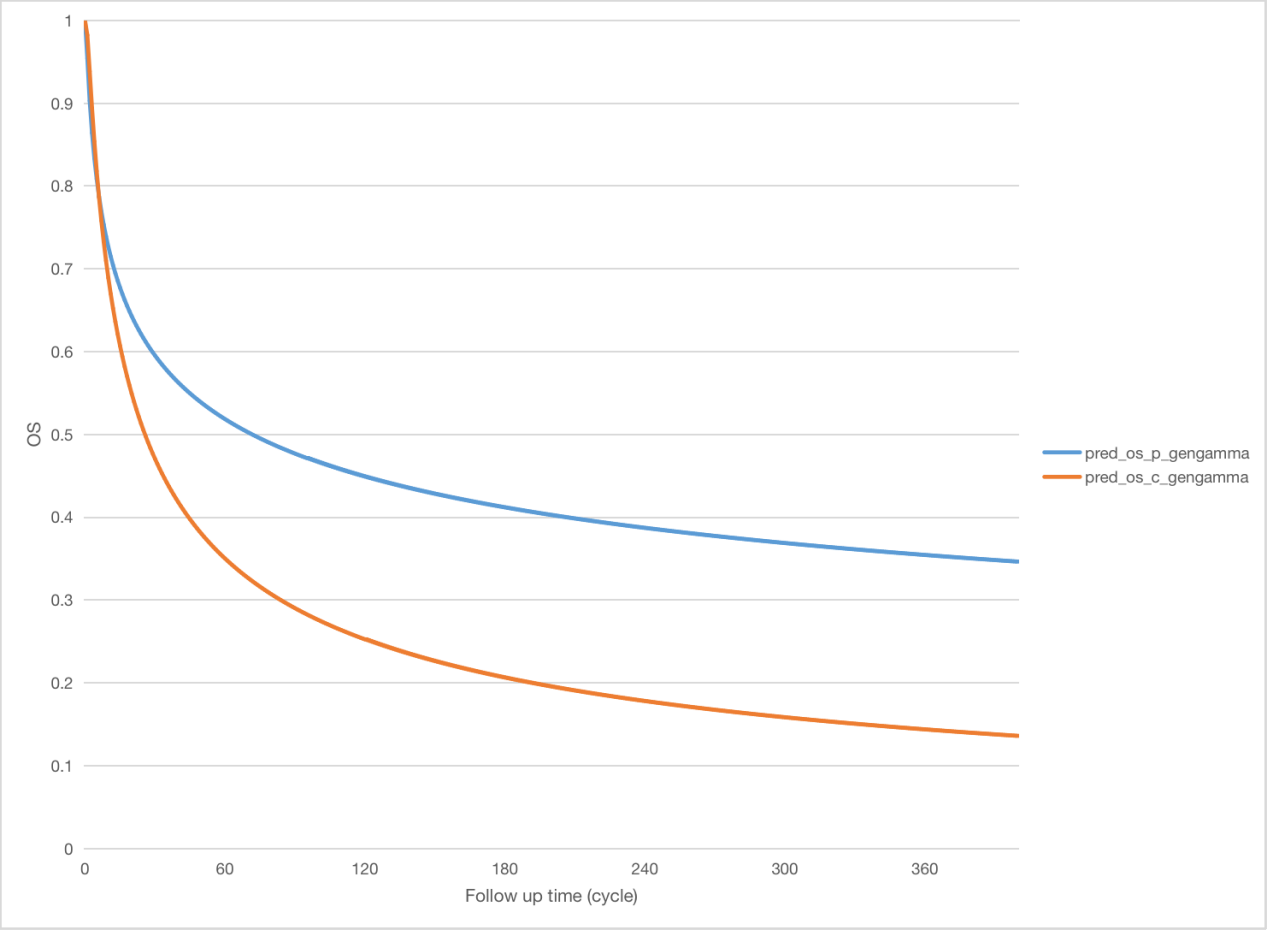


*eFigure 1. Simulated survival curves (OS) for chemotherapy and pembrolizumab*
